# Enhanced Extracellular Matrix Deposition on Titanium Implant Surfaces: Cellular and Molecular Evidences

**DOI:** 10.3390/biomedicines9111710

**Published:** 2021-11-18

**Authors:** Guya Diletta Marconi, Luigia Fonticoli, Ylenia Della Rocca, Stefano Oliva, Thangavelu Soundara Rajan, Oriana Trubiani, Giovanna Murmura, Francesca Diomede, Jacopo Pizzicannella

**Affiliations:** 1 Department of Medical, Oral and Biotechnological Sciences, University “G. d’Annunzio” Chieti-Pescara, 66100 Chieti, Italy; guya.marconi@unich.it; 2 Department of Innovative Technologies in Medicine and Dentistry, University “G. d’Annunzio” Chieti-Pescara, 66100 Chieti, Italy; luigia.fonticoli@unich.it (L.F.); ylenia.dellarocca@unich.it (Y.D.R.); oliva.stefano@hotmail.com (S.O.); oriana.trubiani@unich.it (O.T.); giovanna.murmura@unich.it (G.M.); 3 Department of Biotechnology, Karpagam Academy of Higher Education, Coimbatore 641021, India; drsoundararajan.t@kheduedu.in; 4 Cardiology Intensive Care Unit, “Ss. Annunziata” Hospital, ASL 02 Lanciano-Vasto-Chieti, 66100 Chieti, Italy; jacopo.pizzicannella@unich.it

**Keywords:** osseointegration, osteogenesis, cellular adhesion, gene expression, extracellular matrix (ECM)

## Abstract

The surface structure of the titanium dental implants can modulate the activity of mesenchymal stem cells in order to promote the upregulation of osteoblastic related genes and the release of extracellular matrix (ECM) components. The present work was focused on the in vitro evaluation of the interaction of human periodontal ligament stem cells (hPDLSCs) and two different implant titanium surfaces topography (CTRL and TEST). This study was aimed at analyzing the cytotoxicity of the dental implant surfaces, the cellular adhesion capacity, and the improvement in the release of ECM molecules in an in vitro model. These parameters were carried out by means of the microscopic evaluation, viability assays, immunofluorescence, Western blot and RT-PCR investigations. The knowledge of the cell/implant interaction is essential for implant healing in order to obtain a more performing surfaces that promote the ECM release and provide the starting point to initiate the osseointegration process.

## 1. Introduction

Mesenchymal Stem Cells (MSCs) are well recognized in the regenerative and tissue engineering field for the long-lasting proliferation ability, the capability to differentiate in different lineages, immunomodulation feature and the easy accessibility [1,2,3]. Due to their valuables characteristics and their good ability in tissue and organ regeneration, they have been utilized for clinical studies [4]. The use of periodontal ligament stem cells (PDLSCs) opens a promising possibility for development of tissue engineering technology-based therapeutics approaches for the destroyed periodontium [5].

The periodontium represents the supporting tissues of the teeth which consist of the attachment apparatus and the dento-gingival unit [6]. The human PDLSCs (hPDLSCs) are derived from periodontal ligament, which is characterized by the presence of different cell populations, such as osteoblasts, fibroblasts, epithelial cells, endothelial cells and stem cells [7]. They are considered as a valuable stem cell population for regenerative therapy in periodontium, and they are the widely used MSCs population in the dentistry field [8].

The possibility to obtain the ligament-anchored implants or implants surrounded by periodontal tissue is the gold standard in the dental surgery. The study in the dental implant field has led to a change from smooth machined surfaces to roughened surfaces in order to improve osseointegration thanks to the osteoconductive and osteoinductive properties [9]. The characteristics of the implant surfaces have different implications in the integration that it will be possible to achieve with both hard and soft tissues during rehabilitation. A rough implant allows for a better osseointegration process than a smooth surface.

Previous studies have reported that the cellular interactions with implant surface may promote osseointegration at clinical level [10,11,12]. In particular, the modifications of micro-topography contributed to increase surface area and increased in vivo Bone-to-Implant Contact (BIC) levels [13,14]. Treatment of Ti surface topography altered the growth, metabolism, and migration of osteogenic cells [15,16]. The micro-topography alteration methods, such as sandblasted or acid-etched procedures of the implant surface, have been proposed to increase the osseointegration process at cellular and protein level [17]. At the microscopic level, the biomechanical interlocking between implant and bone can be influenced by the topography of an implant surface [18]. The features of titanium surface may influence the differentiation and morphology of MSCs, the secretion of extracellular matrix (ECM) factors and the osteoblastic gene expressions.

Different findings have demonstrated that a rough surface in comparison with a smooth one promotes a better biomolecular adsorption, augments ECM production and stimulates the differentiation of the MSCs towards an osteoblastic phenotype [19]. Different mechanisms are able to regulate the cell adhesion to a biomaterial. These mechanisms consist of definite interactions between cell surface receptors and specific ligand molecules, which are adsorbed to, placed on, or released over the biomaterial [20]. Adhesion can be establish between adjacent cells (cell-cell adhesion) as well as between cells and the ECM (cell-matrix adhesion). The study of cell adhesion has gained a lot of interest and has been largely investigated in the cellular biology, biomedical and engineering fields [21]. Cell adhesion is involved in inducing signals that modulate cell differentiation, cell cycle, cell migration, and cell survival. It is a fundamental biological event for establishing cell and tissue morphogenesis. This event is modulated by cell adhesion molecules, which are transmembrane receptors connected to the cytoskeleton that permit the assemblage of cells in three-dimensional tissues and their interaction with the surrounding environment [22]. The affinity of cells to substrate is a key factor in biomaterial design and development. The ECM molecules are implicated in the cell-materials interaction events of the implant.

The ECM components in most tissue are categorized into proteoglycans and fibrous proteins such as Fibronectin and Laminin. These molecules give structural support and facilitate cellular communication. Integrins, the transmembrane proteins on the cell surface, connect the cytoskeleton of cells to the ECM, activate signaling pathways that modulate cell proliferation, morphology, adhesion and cell death.

For instance, integrin binding to Fibronectin includes a conformational change in the receptor that results in mechanical coupling to the ligand [23]. Fibronectin glycoprotein interplays with integrins on the cell surface, arbitrates mechanical anchoring and establishes the focal cell–cell formation and cell–material adhesion contacts. The extracellular signals are translated into cellular responses to the areas of cell material adhesion.

In the present work, the expression levels of typical ECM markers, such as Fibronectin, Laminin, Vimentin and N-cadherin were evaluated in an in vitro model of hPDLSCs seeded on two different titanium implant surfaces, CTRL and TEST.

The evaluation of these essential factors may offer an important platform for assessing the biological outcomes of the surface characteristics and the basis for optimizing dental implant surfaces to assist the clinicians when choosing implant systems. Even if the limitation of an in vitro model, the current evidence indicates that SEM and microscopic observations may be useful in providing insight on the role of implant surface topography and the gene expression tests are useful for assessment the cellular and physio-biochemical properties [24]. The findings obtained by the in vitro tests may be correlated to the in vivo studies to better evaluate the surface performances. Thus, changes in micro-topography reflect its effects at a biological level, resulting in the increase of cell adhesion, gene and protein expression related to the formation of ECM, that represent a fundamental step to initiate the osseointegration process.

## 2. Materials and Methods

### 2.1. Ethic Statement

The consent form has been signed by patients enrolled in the present study. The study design has been previously approved by Medical Ethics Committee at the Medical School, “G. d’Annunzio” University, Chieti, Italy (N° 266/17.04.2014). The Department of Innovative Technologies in Medicine and Dentistry and the Laboratory of Stem Cells and Regenerative Medicine are certified according to the quality standard ISO 9001:2008 (N° 32031/15/S).

### 2.2. Cell Culture Establishment

Tissue biopsies were scraped from the horizontal fibers of the periodontal ligament of human premolar teeth. The enrolled patients were in general good health conditions and exempt from oral diseases [25]. The phosphate buffered saline (PBS, Lonza, Basel, Switzerland) solution was used for tissue washing, then the samples were placed in culture with the specific medium for the growth of MSCs, Thera PEAK™MSCGM-CD™ Bullet Kit- chemically defined (MSCGM-CD, Lonza) [26]. Cell culture medium was refresh every 2 or three days. After two weeks of culture, cells spontaneously migrated from the tissue explants and then were sub-cultured till passage 2.

### 2.3. Cell Immunophenotyping

Human PDLSCs at the second passage were collected; 5 × 10^5^ cells were incubated with 1 μg of the specific antibody, conjugated with fluorescein is othiocyanate, phycoerythrin (PE), for 30 min at 4 °C in the dark. Human PDLSCs were stained using the following antibodies: anti-CD14 PE-conjugated (Miltenyi Biotec, Bergisch Gladbach, Germany), anti-CD34 PE-conjugated (Beckman Coulter, Fullerton, CA, USA), anti-CD73 PE-conjugated, anti-CD90 FITC-conjugated (Becton Dickinson, San Jose, CA, USA) and anti-CD105 (Ancell, Stillwater, MN, USA). After incubation, cells were acquired with a flow cytometer (FACS Calibur, BD Bioscience, Franklin Lakes, NJ, USA). Data were analyzed by the FlowJo software (v8.8.6, TreeStar, Ashland, OR, USA).

### 2.4. Mesengenic Differentiation and Histochemical Analysis

To evaluate the capacity of hPDLSCs to differentiate into adipogenic and osteogenic lineages, cells at passage 2 were used. For adipogenic differentiation expanded cultured with MSCGM-CD, were seeded into dishes at a density of 2 × 10^4^ cells/cm^2^. At 100% confluence, three cycles of induction/maintenance stimulated adipogenic differentiation. Each cycle consisted of feeding the hPDLSCs with supplemented adipogenesis induction medium (Lonza) for 3 days, followed by 3 days in supplemented adipogenic maintenance medium (Lonza). After three complete cycles of induction/maintenance, the culture was incubated for 7 days in supplemented adipogenic maintenance medium, replacing the medium every 2–3 days. The cells were fixed in 10% formalin for 15 min and washed with dH_2_O. Subsequently, the cells were stained with Oil Red O (ORO) working solution (300 mg of Oil red O/100 mL of isopropanol) for 5 min and counterstained with hematoxylin. For osteogenic induction, the primary cells at the second passage were seeded at 4 × 10^3^ cells/cm^2^ in MSCGM-CD culture medium and maintained in culture at 37 °C, in a humidified 5% CO_2_ atmosphere. At sub-confluence, cells were incubated with MSCGM-CD medium with the addition of osteogenic supplements, as 100 nM dexamethasone (Applichem, Darmstadt, Germany), 10 nM bglycerol-phosphate (Applichem), and 0.05 mM 2-phosphateascorbicacid (Sigma-Aldrich, Milan, Italy). Visualization of calcium deposition and ECM mineralization was obtained by Alizarin Red S (ARS) staining assay performed after 21 days of culture. According to Gregory et al., 32 cells were washed with PBS, fixed in 10% (*v*/*v*) formaldehyde (Sigma-Aldrich) for 30 min, and washed twice with abundant dH_2_O before addition of 0.5% Alizarin red S in H_2_O, pH 4.0, for 1 h at RT. After cell incubation under gentle shaking, cells were washed with dH_2_O four times for 5 min and observed under light microscopy.

### 2.5. Dental Implants

In the present work, two titanium disks surfaces (Resista, Omegna, VB, Italy) have been used: sandblasted (CTRL) and dual acid etched (TEST). The disks were made of grade 4 titanium [27]. In particular, the TEST surface was obtained through the non-contaminant micro-subtraction process, Double Acid Etching (DAE), changes the implant micro roughness and the surface texture maximizing the wettability and biomimetic properties.

### 2.6. Exposure to the Titanium Surfaces

Human PDLSCs were seeded on titanium disks, CTRL and TEST, with MSCGM-CD (Lonza). The culture medium was changed twice a week. Cultures were maintained for 8 weeks, then were processed as described below for further examination. The inverted light microscopy DMIL (Leica microsystem, Milan, Italy) was used to carried out the microphotographs in order to evaluate the morphological arrangement of hPDLSCs and titanium disks.

### 2.7. Cell Viability Assay

The cell metabolic activity of hPDLSCs seeded with or without CTRL and TESTsamples was measured using the 3-(4,5-dimethylthiazol-2-yl)-5-(3-carboxymethoxyphenyl)-2-(4-sulfo-phenyl)-2H-tetrazolium (MTS) assay (CellTiter 96^®^ Aqueous One Solution Cell Proliferation Assay, Promega, Madison, WI, USA). Briefly, the cells were seeded into 96-well plates with MSCBM-CD medium (Lonza) at 2400 cells/well for 24, 48 and 72 h and 1 week at 37 °C. After the different time points 15 μL/well of MTS dye solution was added to culture medium, and cells were incubated for 3 h at 37 °C. The quantity of formazan product, directly proportional to the number of living cells in culture, was detected by absorbance measurements at 490 nM wavelength utilizing the Synergy™ HT Multi-detection microplate reader (Biotech, Winooski, VT, USA). The MTS assay was executed in three independent experiments.

### 2.8. Scanning Electron Microscopy (SEM) Analysis

SEM analyses were then performed to evaluate the relationship between hPDLSCs and the titanium disc surfaces.

The samples for SEM analysis were fixed for 4 h at 4 °C in 4% Glutaraldehyde in 0.05 M PBS (pH 7.4) and then dehydrated in increased range of ethanol concentrations and dried out Specimens were mounted on aluminum stubs and gold-coated in an Emitech K550 sputter-coater (Emitech Ltd., Ashford, UK). SEM EVO 50 (Zeiss, Jena, Germany) was used for analysis [28].

### 2.9. Confocal Laser Scanning Microscopy (CLSM) Analysis

The hPDLSCs were seeded on CTRL and TEST titanium disks surfaces for 8 weeks, the medium was replaced every 2 days, and then were fixed for 10 min at room temperature (RT) with 4% paraformaldehyde in 0.1 M PBS (pH 7.4). After washing procedure samples were processed for the immunofluorescence staining. The specimens were permeabilized with 0.5% Triton X-100 in PBS, followed by blocking with 5% skimmed milk in PBS. Primary monoclonal antibodies to anti-human Fibronectin (antibody dilution 1:200) (sc-8422, Santa Cruz Biotechnology, Santa Cruz, CA, USA), Laminin (antibody dilution 1:200) (sc-17810, Santa Cruz Biotechnology), N-cadherin (sc-59987, Santa Cruz Biotechnology) and RUNX2 (sc-399351, Santa Cruz Biotechnology) were used, followed by Alexa Fluor 488 green fluorescence conjugated goat anti-mouse as secondary antibodies (cat. n. A 11029, Molecular Probes, Eugene, OR, USA). Then, Alexa Fluor 594 phalloidin red fluorescence conjugate (antibody dilution 1:200) (cat. n. A12381, Molecular Probes) was used to highlight the cytoskeleton actin and TOPRO (dye dilution 1:200) (cat. n. T3606, Molecular Probes) was used to stain the cell nuclei [29]. LSM800 META (Zeiss) confocal system was used to evaluate the specific markers expression. The confocal system was connected to an inverted Zeiss Axiovert 200 microscope equipped with a Plan Neofluar oil-immersion objective (40×/1.3 NA, Zeiss).

### 2.10. RNA Isolation and Real-Time RT-PCR Analysis

RUNX2, VIM, FN1, CDH2, LAMB1, ITGA5 and ITGB1 mRNA expression were analyzed by real-time PCR. Total RNA was extracted using PureLink RNA Mini Kit (Ambion, Thermo Fisher Scientific, Milan, Italy) according to the manufacturer’s instructions.

Three independent biological replicates were analyzed for each sample. Two micrograms of total RNA were retrotranscribed using the High Capacity cDNA Reverse Transcription kit catalog number 4,368,814 (Applied Biosystems, Waltham, MA, USA) to synthesize cDNA for 10 min at 25 °C, 10 min at 37 °C and 5 min at 85 °C according to the manufacturer instructions.

Real-Time PCR was performed with Mastercycler ep real plex real-time PCR system (Eppendorf, Hamburg, Germany). The levels of mRNA expression of RUNX2, FN1, CDH2, LAMB1, ITGA5, ITGB1 and Beta-2 microglobulin (B2M) (endogenous marker) (Table 1) were evaluated in hPDLSCs cells cultured on CTRL compared to the mRNA expression levels of hPDLSCs cultured on TEST titanium disk. Commercially available TaqMan Gene Expression Assays (RUNX2 Hs00231692_m1; VIM Hs.PT.58.38906895; FN1 Hs.PT.58.40005963; CDH2 Hs.PT.58.26024443; LAMB1 Hs.PT.58.3739165; ITGA5 Hs.PT58.4796384 and ITGB1 Hs.PT.58.39883300) and the TaqMan Universal PCR Master Mix (Applied Biosystems, Foster City, CA, USA) were utilized according to standard protocols [30]. Beta-2 microglobulin (B2M Hs99999907_m1) was utilized for template normalization. The amplification program consisted of a preincubation step for cDNA denaturation (3 min 95 °C), followed by 40 cycles consisting of a denaturation step (15 s 95 °C), and an annealing step (1 min 60 °C). At the end of each run, melting curve was performed in the temperature range of 60 to 95 °C. Expression levels for each gene were performed according to the 2^−ΔΔCt^ method. RT-PCR was performed in three independent experiments; duplicate determinations were performed for each specimen.

### 2.11. Protein Expression

The cell lysates (50 µg) underwent electrophoresis and were transferred to the polivinilidenfluoruro (PVDF) membrane. The latter were blocked in 5% of non-fat milk in PBS 0.1% Tween-20, then membranes were incubated with primary antibodies anti-human Fibronectin (antibody dilution 1:750) (sc-8422, Santa Cruz Biotechnology), anti-Laminin (antibody dilution 1:750) (sc-17810, Santa Cruz Biotechnology), N-cadherin (antibody dilution 1:750) (sc-59987, Santa Cruz Biotechnology), anti-RUNX2 (antibody dilution 1:500) (sc-390351, Santa Cruz Biotechnology), anti-OPN (antibody dilution 1:350) (sc-21742, Santa Cruz Biotechnology), anti-SPARC (antibody dilution 1:350) (sc-25574, Santa Cruz Biotechnology) and anti-beta-actin (antibody dilution 1:750) (sc-69879, Santa Cruz Biotechnology). After five washes in PBS (Lonza) containing 0.1% Tween-20, membranes were incubated for 1 h at room temperature with peroxidase-conjugated secondary antibody anti-mouse (A90-116P Goat anti-mouse, Abcam, Milan, Italy) 1:2000 diluted in 1X PBS (Lonza), 3% milk, and 0.1% Tween-20 [31]. Protein expression was visualized using the enhanced chemiluminescence detection method (ECL) (Amersham Pharmacia Biotech, Milan, Italy) with photo documenter Alliance 2.7 (Uvitec, Cambridge, UK). Signals were analyzed by ECL enhancing and evaluated through UVIband-1D gel analysis (v2.7, 1D Max software, Uvitec). Data were normalized with densitometric values derived from β-actin loading control.

### 2.12. Data and Statistical Analysis

The Statistical Package for Social Science (SPSS Inc., v.21.0, Chicago, IL, USA) was used for data analysis. Data were expressed as means and standard deviation of the recorded values. The differences among the levels of the factors under investigation were analyzed performing three distinct two-way-ANOVA tests, one for each experiment. Tukey tests were applied for pairwise comparisons. A value of *p* < 0.05 was considered statistically significant in all tests. Regarding gene expression analysis, the comparative 2^−ΔΔCt^ method was used to quantify the relative abundance of mRNA and then to determine the relative changes in individual gene expression (relative quantification) [32].

## 3. Results

### 3.1. Cell Characterization

**Figure 1:**
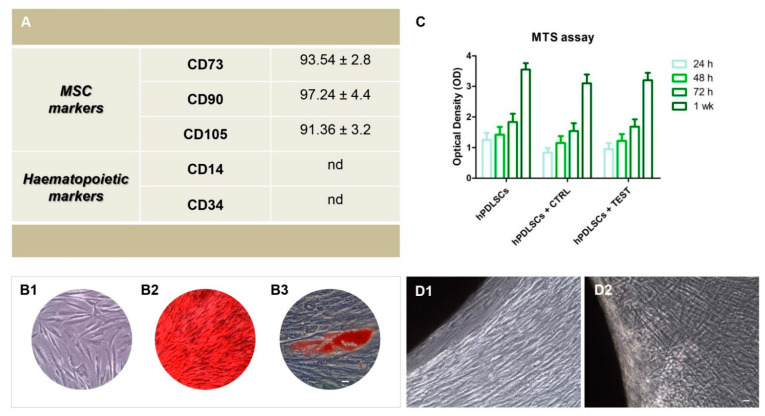
(**A**) Cytofluorimetric analysis of hPDLSCs primary culture at second passage. The values are expressed as mean fluorescence ratio (MFI) obtained by dividing the MFI of positive events by the MFI of negative events. (**B1**) Plastic adherent primary cultures of hPDLSCs observed at light microscopy showed a spindle-shaped appearance with long cytoplasmatic processes. (**B2**) The adipogenic potential of hPDLSCs grown was analyzed through the evaluation of intracellular lipid droplets by light microscopy using Oil Red O staining after adipogenic induction. (**B3**) The osteogenic differentiation was evaluated by staining with Alizarin red S (ARS) that demonstrated the calcium deposition. (**C**) Cell viability assay at 24, 48, 72 and 1 week of culture. (**D1**) hPDLSCs cultured with CTRL surface. (**D2**) hPDLSCs cultured with TEST surface. Scale bar: 20 µm.

Cytofluorimetric experiments showed the expression of surface molecules as CD73, CD90 and CD105. The hPDLSCs were negative for the subsequent markers CD14, CD34 (Figure 1A). To evaluate the morphological features of hPDLSCs the primary cultures were observed by means of inverted light microscopy. Adherent cells on glass cover slips showed a spindle-shaped morphology with elongated cytoplasmic processes (Figure 1B1). Alizarin Red S staining was performed to visualize the calcium deposits. To evaluate the adipogenic differentiation of hPDLSCs the cellular monolayer was stained with Oil Red O and observed by light microscopy. Adipogenic-induced cells showed the well evident intracellular lipid droplets as single or grouped (Figure 1B2). The hPDLSCs showed appositive staining for Alizarin Red S solution and that several areas with high levels of mineralization related to bone nodule were evident (Figure 1B3).

### 3.2. Cell Viabilityassay and Morphological Features of hPDLSCs Cultured on CTRL and TEST Titanium Surfaces

MTS assay was performed at 24, 48, 72 h, and 1 week on hPDLSCs alone and seeded on CTRL and TEST titanium disk surfaces. The hPDLSCs cultured on the CTRL and TEST disk exhibited a similar viability rate compared to the hPDLSCs at different considered time points. The cell viability rate follows a logarithmic trend (Figure 1C). Microphotographs obtained from inverted light microscopy showed the cell adhesion to both type of titanium disks, CTRL and TEST; in particular, on TEST surface cells showed a better arrangement and adherence capacity around the disk surface (Figure 1D1,D2).

### 3.3. hPDLSCs Adhesion

**Figure 2:**
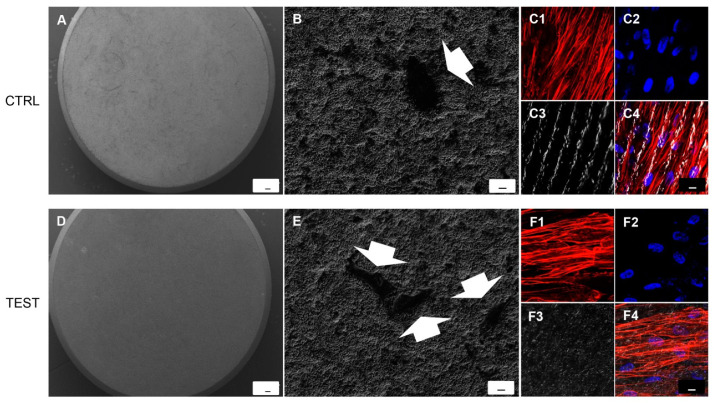
SEM analysis showed the hPDLSCs adhesion capacity to the titanium implant surfaces. (**A**) CTRL surface without cells. (**B**) hPDLSCs adhered on CTRL surface. (**D**) TEST surface without cells. (**E**) hPDLSCs adhered on TEST surface. Arrows indicate the cellular profile. Mag: (**A**,**D**) 50×; (**B**,**E**) 1000×. Scale bars: (**A**,**D**) 100 µm; (**B**,**E**) 10 µm. (**C1**–**C4**) Immunofluorescence images of hPDLSCs cultured on CTRL titanium implant surface. (**F1**–**F4**) Immunofluorescence images of hPDLSCs cultured on TEST titanium implant surface. Mag: 40×. Scale bar: 10 µm.

SEM observations evidenced the surface morphology of two different surfaces, CTRL and TEST without cells (Figure 2A,D). Human PDLSCs seeded on TEST surface for 24 h showed a better ability of adhesion compared to CTRL surface (Figure 2B,E). To better evaluate the adhesion capacity and cytoskeleton arrangement at intracellular level, the immunofluorescence experiments have been performed. Cells cultured on CTRL and TEST surfaces showed the presence of evident actin fibers stained in red (Figure 2C1–C4). On TEST surface cells were distributed in a multilayer way evidencing a better performance of the treated surface (Figure 2F1–F4).

### 3.4. Implant Titanium Disks Affect Protein Expression at CLSM

**Figure 3:**
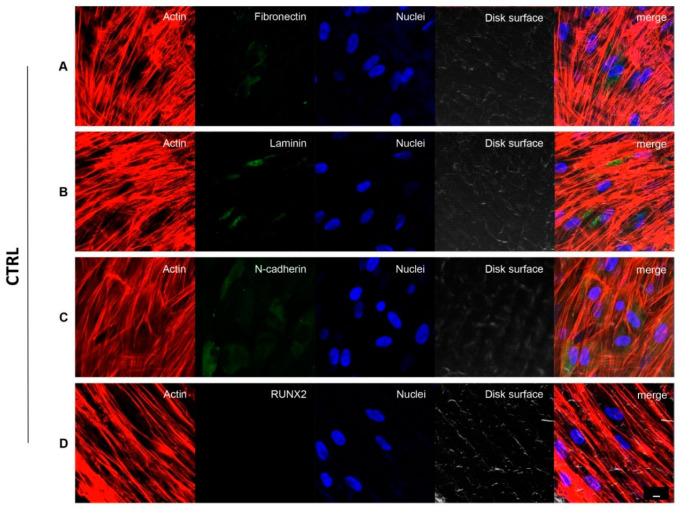
hPDLSCs cultured on CTRL titanium implant surface observed after 8 weeks. Cytoskeleton actin was stained in red fluorescence; specific markers:Fibronectin (**A**), Laminin (**B**), N-cadherin (**C**) and RUNX2 (**D**) were stained in green fluorescence; nuclei were stained in blue fluorescence. Scale bar: 10 µm.

**Figure 4:**
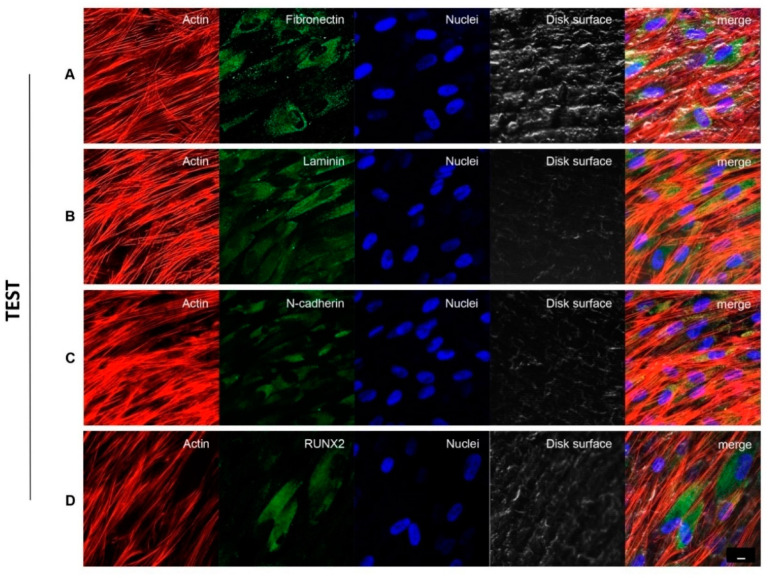
hPDLSCs cultured on TEST titanium implant surface observed after 8 weeks. Cytoskeleton actin was stained in red fluorescence; specific markers: Fibronectin (**A**), Laminin (**B**), N-cadherin (**C**) and RUNX2 (**D**) were stained in green fluorescence; nuclei were stained in blue fluorescence. Scale bar: 10 µm.

Figure 3 and Figure 4 showed fluorescence images of the cytoskeleton actin (phalloidin, green) and the nuclei (TOPRO, blue) of hPDLSCs seeded on CTRL and TEST specimens captured after 8 weeks of culture. The cells adhered and spread well with a spindle fibroblast-like shape on both samples which revealed that the different surface treatment did not affect the adhesion capability. The CLSM observation showed a higher RUNX2 expression level for hPDLSCs seeded on TEST compared to CTRL, suggesting a better capability of hPDLSCs seeded on TEST surface to differentiate towards the osteogenic lineage after 8 weeks of culture (Figure 3 and Figure 4). Furthermore, hPDLSCs cultured on TEST implant surface evidenced a higher expression of Fibronectin, Laminin and N-cadherin compared to cells seeded on CTRL after 8 weeks (Figure 3 and Figure 4).

### 3.5. Genes and Proteins Expression

**Figure 5:**
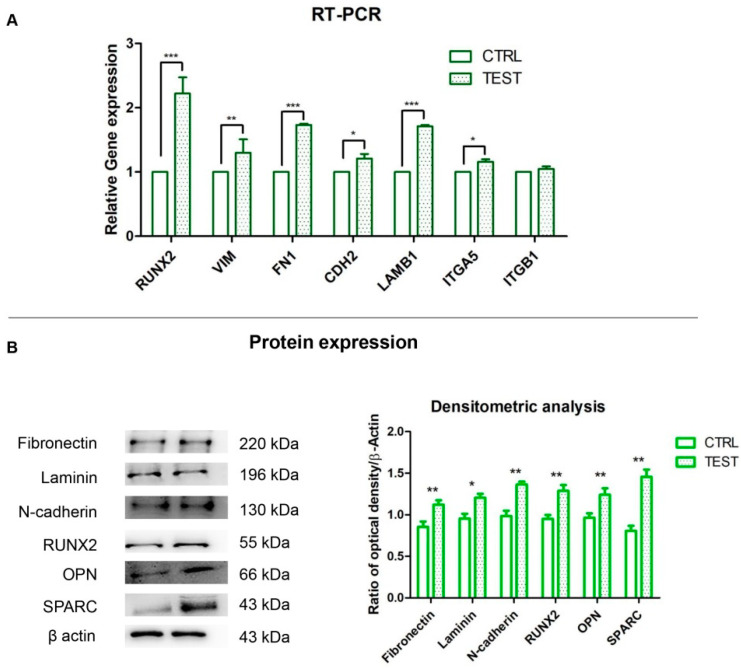
(**A**) Histograms of RT-PCR showed the mRNA levels of RUNX2, VIM, FN1, CDH2, LAMB1, ITGA5 and ITGB1 in hPDLSCs cultured on CTRL and TEST surface * *p* < 0.05; ** *p* < 0.01; *** *p* < 0.001. (**B**) Protein expression of Fibronectin, Laminin, N-cadherin, RUNX2, OPN and SPARC in hPDLSCs cultured on CTRL and TEST surface. Histograms represent densitometric measurements of proteins bands expressed as integrated optical intensity with the mean of three separate experiments. The error bars showed standard deviation (±SD). Densitometric values analyzed by ANOVA showed significant differences. * *p* < 0.05; ** *p* < 0.01.

The histogram indicated the gene expression of RUNX2, VIM, FN1, CDH2, LAMB1, ITGA5 and ITGB1 evaluated by RT-PCR (Figure 5A). The hPDLSCs seeded on TEST reported a significant higher expression of RUNX2 in comparison with hPDLSCs seeded on CTRL disk surface. Moreover, hPDLSCs cultured on TEST surface evidenced a significant higher gene expression of VIM, FN1 and LAMB1 compared to the CTRL surface, and a slight increase in relative gene expression level of CDH2 and ITGA5 in TEST disk in comparison with CTRL surface. Conversely, no significant difference was noticed in the gene expression levels of ITGB1 in hPDLSCs on CTRL and TEST titanium disk. Gene expression confirmed the qualitative results obtained by CLSM observations. Densitometric analysis of the relative protein expression of RUNX2, N-cadherin, Fibronectin, Laminin, OPN and SPARC showed an over expression in hPDLSCs cultured on TEST compared to the cells seeded on CTRL surface (Figure 5B). Moreover, the densitometric analysis of protein levels showed a high expression of ECM proteins which validated the immunofluorescence results (Figure 5B).

## 4. Discussion

Nowadays, re-establishing the oral health using dental implants is considered the gold standard treatment in oral rehabilitation of partially or fully edentulous patients. The success of dental implant engraftment is to develop a structural and functional connection between newly formed bone and the implant surface in order to obtain the biomechanical stability [33]. The osseointegration process is a complex physiological mechanism that starts from ECM deposition leading to bone tissue formation through distinct phases of implant site healing. Lately, a lot of scientists put their efforts on the development of biomaterials with specific characteristics which enable them to circumvent the failures of dental implant. The early step of osseointegration starts with cell adhesion and differentiation and the dental implant surface topography represents a key factor to the initial phase as well as in long-term bone remodeling [34,35].

Based on the literature, several treatment methods have been performed on Ti implant to promote the optimal interaction between the bone and the implant surface to ensure faster rehabilitation of the patients [36]. Several methods are available to modify the implant surface, such as physicochemical, morphological, and biochemical techniques [37]. The treatment methods, namely sandblasting and acid etching, are considered to be able to obtain high performing surfaces. Sandblasting is the bombardment of titanium surface with granules of variable diameter of oxides (titanium dioxide, aluminum oxide, zirconium dioxide and silicon carbide). Instead, acid etching method is performed with sulfuric, hydrofluoric or hydrochloric acid as stated by different procedures [38]. Surface features can play an important role in the biomolecular adsorption and cell adhesion to the implant surface as well as in osteoblast cell maturation [39,40,41].

MSCs can be simply isolated from craniofacial tissues during dentistry procedures. These cells exemplify as appropriate cell populations to study cell-biomaterial interaction in the craniofacial field, including osteointegrative events [42]. MSCs have the capacity of self-renewal and differentiation into many cell lineages that comprise osteoblasts, chondrocytes, adipocytes, neuroblasts and myoblasts. In our in vitro cellular model, we use hPDLSCs, which are autologous and easily obtainable cell populations, showing the essential features for human clinical use [43,44]. In particular, hPDLSCs represent a suitable model for the study of bone differentiation, thanks to their osteogenic capacity, compared to other types of MSCs.

In the present study we evaluated the secretion of ECM components necessary for osseointegration and osteogenesis using an in vitro model of hPDLSCs cultured on two different Ti surfaces, CTRL and TEST. The morphology and the cell adhesion capability of hPDLSCs on CTRL and TEST titanium surfaces were analyzed by microscopic analysis. The hPDLSCs evidenced similar morphology and adhesion capability on both CTRL and TEST Ti implant surfaces. In our study, the expression levels of principal and typical markers of ECM Fibronectin and Laminin, cytoskeleton markers actin and Vimentin, N-cadherin, a key factor in cell-cell interaction, and RUNX2, an early osteogenic marker were analyzed by Confocal laser scanning microscopy (CLSM) and RT-PCR. The results obtained from CLSM observation and RT-PCR revealed a significant upregulation of Fibronectin, Laminin, N-cadherin and RUNX2 in hPDLSCs seeded on TEST compared to CTRL surface. These results highlighted that the TEST surface induced the release of ECM matrix components fibronectin and Laminin which may promote hPDLSCs to undergo osteogenesis and implant osseointegration as evidenced from the higher expression of RUNX2 in TEST with respect to CTRL sample.

Furthermore, the slight increase of the expression level of N-cadherin could contribute to the enhancement of hPDLSCs osteogenesis, thus lead to more bone matrix deposition on the Ti implant. In parallel, these results were confirmed by the data obtained by Western Blot, which showed the quantitative level of protein expression of Fibronectin, Laminin, N-cadherin, RUNX2, OPN and SPARC. It is well known that ECM regulates cell adhesion. Cells adhere to the ECM via an integrin-mediated adhesion that connects ECM to the cytoskeleton, and integrins serve as receptors for ECM proteins [45]. In order to ameliorate the regeneration, it is essential to evaluate the cell binding strength to the implant material surface [46]. In particular, some expressed proteins are vital for the osteogenesis process, RUNX2 is essential and required for physiologic bone formation, osteopontin is a protein involved in normal calcifications and SPARC is one of the most abundant non-collagenous proteins expressed in the mineralized tissues [47]. The evaluation of molecules such as integrins in the process of cellular attachment is needed to develop a Ti surface with optimum adherence properties for periodontal ligament [48]. On this basis, in our study the gene expression level of RUNX2, VIM, CDH2, LAMB1, ITGA5 and ITGB1 was investigated. ITGA5 plays a fundamental role in osteogenic commitment as it is expressed by osteoblasts and osteoprogenitors and stimulates cell survival and matrix mineralization. ITGB1 is stably expressed by osteoblasts during different stages of osteogenesis, and it also mediates cell attachment to fibronectin as well as fibronectin assembly. Integrin-fibronectin interactions are vital for important cellular behaviors including embryogenesis, wound healing and cell migration [49].

In the current work, the authors demonstrated how rough titanium surfaces, obtained by double acid attack, improves the ECM components production and the expression of osteoblastic cellular profile, in order to promote the bone generation, anchorage, and osteointegration of titanium-made dental implants.

Surface modifications on the implant surfaces have been designed to improve the performance of the dental implant, in particular the roughness is considered a key factor for primary and long-term implant stability. This study was conducted to test the ECM molecules production. The ECM formation represents the early stage in the osteogenesis process, which encourages the cell migration, proliferation and differentiation, thus reducing the surgical time and morbidity of implant site via enhancing the bone tissue formation.

## 5. Conclusions

To conclude, the present results have shown a higher-level expression of typical markers of ECM Laminin and Fibronectin and its receptor Integrin α-5, and an upregulation of in cell-cell interaction marker N-cadherin, suggesting a better performance of TEST surface compared to CTRL in terms of osteogenesis and implant osseointegration as evidenced from the higher expression of RUNX2 in TEST respect to CTRL sample. Future research should examine the advancements in dental implant surface design, which are crucial to improve the healing process and to enhance the bone formation in order to permit the use of early loading protocols and to guarantee the implant success in patients with compromised bone tissue.

## Figures and Tables

**Table 1:** Primer sequences used for real-time PCR reactions.

Gene	Forward Primer Sequence (5-3)	Reverse Primer Sequence (5-3)
**RUNX2**	CTTCACAAATCCTCCCCAAGT	AGGCGGTCAGAGAACAAAC
**VIM**	CAAGACCTGCTCAATGTTAAGATG	GTGAATCCAGATTAGTTTCCCTCA
**FN1**	CGTCCTAAAGACTCCATGATCTG	ACCAATCTTGTAGGACTGACC
**CDH2**	GTTTGCCAGTGTGACTCCA	CATACCACAAACATCAGCACAAG
**LAMB1**	TTG GAG CAA ATG TAG TGA CCA	CTA CTG TAT CGT CAG CCA CTT G
**ITGA5**	ACCAACAAGAGAGCCAAAGTC	TTGTACACAGCCTCACACTG
**ITGB1**	GTAGCAAAGGAACAGCAGAGA	GGTCAATGGGATAGTCTTCAGC

## Data Availability

Data are available upon request.
